# Endogenous T Cell Receptor Rearrangement Represses Aggressive Central Nervous System Autoimmunity in a TcR-Transgenic Model on the Non-Obese Diabetic Background

**DOI:** 10.3389/fimmu.2019.03115

**Published:** 2020-01-15

**Authors:** Asmita Pradeep Yeola, Prenitha Mercy Ignatius Arokia Doss, Joanie Baillargeon, Irshad Akbar, Benoit Mailhot, Mohammad Balood, Sébastien Talbot, Ana Carrizosa Anderson, Steve Lacroix, Manu Rangachari

**Affiliations:** ^1^Axe Neurosciences, Centre de Recherche du CHU de Québec—Université Laval, Quebec City, QC, Canada; ^2^Department of Physiology and Pharmacology, Université de Montréal, Montréal, QC, Canada; ^3^Evergrande Center for Immunologic Diseases and Ann Romney Center for Neurologic Diseases, Harvard Medical School and Brigham and Women's Hospital, Boston, MA, United States; ^4^Department of Molecular Medicine, Faculty of Medicine, Laval University, Quebec City, QC, Canada

**Keywords:** EAE (experimental autoimmune encephalomyelitis), 1C6, TCR transgenic mice, Treg—regulatory T cell, non-obese diabetic (NOD), allelic exclusion, RAG, FoxP3

## Abstract

The T cell response to central nervous system (CNS) antigen in experimental autoimmune encephalomyelitis (EAE) permits one to model the immune aspects of multiple sclerosis. 1C6 transgenic mice on the non-obese diabetic (NOD) background possess a class II-restricted T cell receptor (TcR; Vα5-Vβ7) specific for the encephalitogenic peptide myelin oligodendrocyte glycoprotein (MOG)_[35−55]_. It remains to be determined what role is played by allelic inclusion in shaping the TcR repertoire of these mice. Here, we show that 1C6 T cells display substantial promiscuity in their expression of non-transgenically derived Vα chains. Further, enforced expression of the transgenic TcR in 1C6 × *Rag1*^−/−^ mice profoundly disrupted thymic negative selection and led to a sharp decrease in the number of mature peripheral T cells. 1C6 × *Rag1*^−/−^ mice developed spontaneous EAE at a significant frequency and rapidly developed fatal EAE upon immunization with myelin oligodendrocyte glycoprotein (MOG)_[35−55]_. Passive transfer of 1C6 × *Rag1*^+/+^ CD4^+^ T cells, but not CD8^+^ T cells or B cells, partially rescued 1C6 × *Rag1*^−/−^ mice from severe EAE. FoxP3^+^ CD4^+^ T_reg_ cells were present in the CNS of immunized 1C6 mice, as well as immunized 1C6 × *Rag1*^−/−^ that had been supplemented with 1C6 CD4^+^ T cells. However, they were not observed in 1C6 × *Rag1*^−/−^ that did not receive Rag1-sufficient 1C6 CD4^+^. Further, *in vivo* blockade of T_reg_ accelerated the onset of symptoms in 1C6 mice immunized with MOG_[35−55]_, indicating the pertinence of T_reg_-mediated control of autoimmune inflammation in this model. Thus, TcR allelic inclusion is crucial to the generation of FoxP3^+^ CD4^+^ T cells necessary for the suppression of severe CNS autoimmunity.

## Introduction

Multiple sclerosis (MS) is a chronic degenerative disease of the brain, spinal cord and optic nerve that affects an estimated 2 million people worldwide. It is generally accepted that MS is driven by self-reactive adaptive immune responses to central nervous system (CNS) components such as myelin. Accordingly, the majority of current MS therapies target the adaptive immune system (1, 2). The contributions of the immune system to MS pathology can be in large part recapitulated by the animal model, experimental autoimmune encephalomyelitis (EAE) (3). In classic EAE, mice of genetically susceptible backgrounds are immunized with CNS-derived (most frequently myelin-derived) encephalitogenic epitopes. In the past two decades, the introduction of T cell receptor-transgenic (TcR-Tg) lines, in which mice possess T cells with transgenically-encoded antigen specificity for encephalitogenic epitopes, has furthered our understanding of the role played by T cells in CNS autoimmunity (4).

When actively immunized with myelin oligodendrocyte glycoprotein (MOG)_[35−55]_, non-obese diabetic (NOD) background mice present a relatively slow-developing disease over 70–100 days that is characterized by multiple relapses and remissions followed by chronically worsening symptoms, thus recapitulating the most common form of MS. This has led some to characterize NOD-EAE as a model of secondary-progressive (SP) MS (5–7). Recently an MHC class II-restricted, MOG_[35−55]_-specific TcR-Tg strain, 1C6, was described on the NOD background. The 1C6 TcR α-chain incorporates Vα5 and the 1C6 TcR β-chain incorporates Vβ7 (8). Despite being class II-restricted, the 1C6 TcR is expressed on both CD4^+^ and CD8^+^ T cells. Interestingly, a proportion of 1C6 T cells express alternate Vα chains (8), in line with previous observations that expression of an ectopic TcR does not exclude the arrangement and expression of a TcR from endogenous alleles (9).

Here, to examine the consequences of enforced allelic exclusion in the 1C6 strain, we studied 1C6 × *Rag1*^−/−^ mice, which are incapable of rearranging their endogenous TcRα and β chains. We found that naïve 1C6 × *Rag1*^−/−^ mice have diminished numbers of both CD4^+^ and CD8^+^ T cells. Intriguingly, 1C6 × *Rag1*^−/−^ mice developed extremely severe EAE upon MOG_[35−55]_ immunization. This severe effect could be partially rescued by the infusion of Rag1-sufficient CD4^+^, but not CD8^+^, 1C6 T cells. We found that FoxP3^+^ CD4^+^ T cells were largely absent from the CNS of 1C6 mice of immunized 1C6 × *Rag1*^−/−^ mice, and that infusion of Rag1-sufficient 1C6 CD4^+^ T cells rescued this population. Overall, we found that endogenously rearranged TcR chains are essential in mediating the development of regulatory T cells that determine disease susceptibility in 1C6 mice.

## Results

### Mature T Cell Survival in 1C6 Mice Requires Endogenous TcR Rearrangement

**Figure 1:**
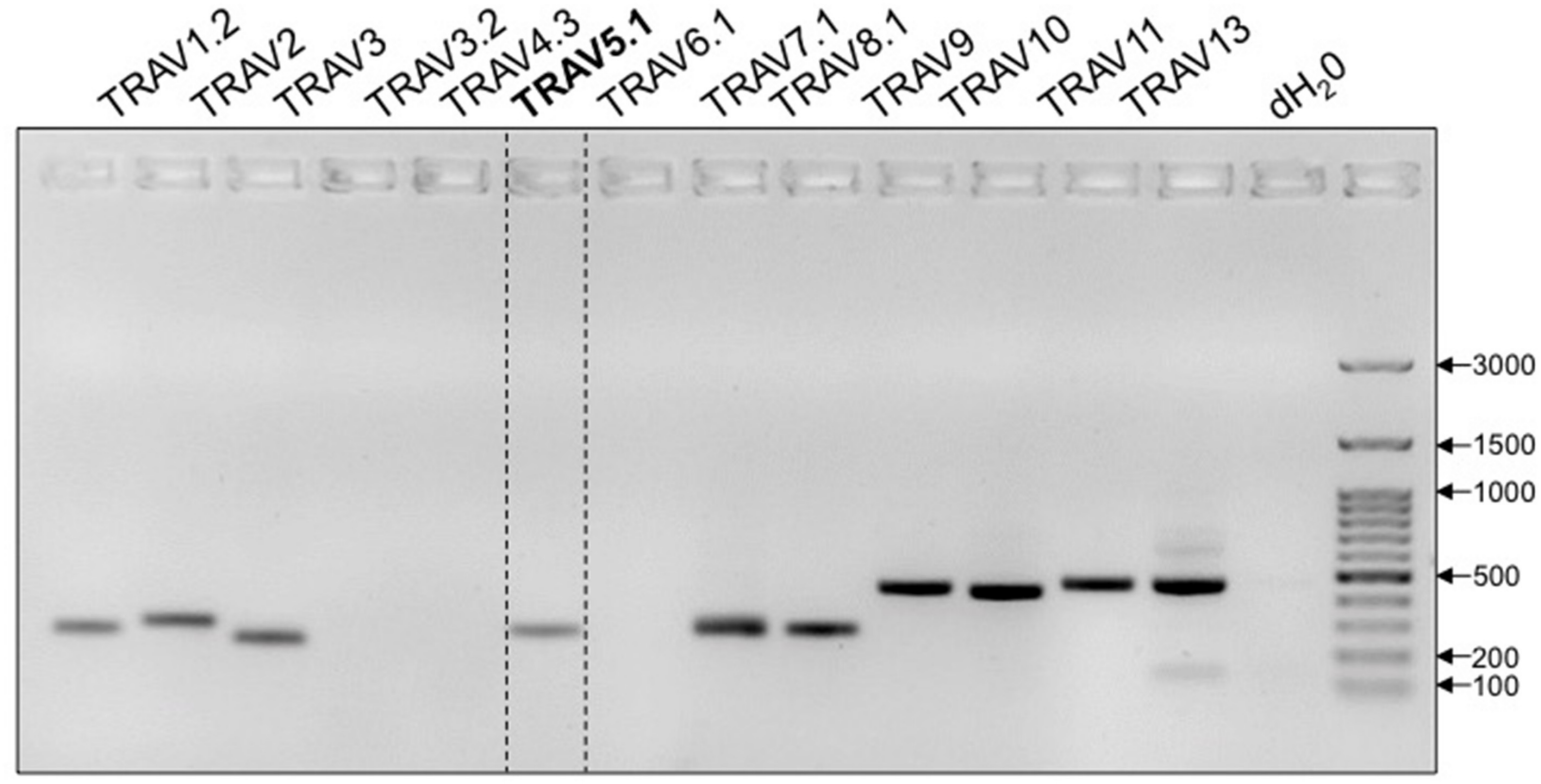
1C6 Tg T cells are promiscuous in their expression of endogenous TcRα. Purified splenic 1C6 CD8^+^ T cells were assessed for the presence of specific TcRα DNA by PCR. Transgenically encoded Vα5.1 bracketed by dotted lines. Representative of three experiments.

TcR development in the thymus entails the rearrangement and ligation of distinct α-chain V(ariable) and J(oining) segments, or β-chain V, D(iversity) and J segments. Excluded segments are lost from the genome of the cell. While the vast majority of 1C6 T cells express the transgenic TcR (8), it has been previously observed that allelic exclusion in TcR-Tg strains is incomplete (9, 10). We therefore wanted to comprehensively assess the expression of non-transgenic, endogenously-derived Vα segments in 1C6 T cells. Due to the paucity of commercially available flow cytometry antibodies to specific Vα alleles, we employed a PCR-based approach. We found that in addition to transgenic Vα5, 1C6 CD8^+^ T cells possessed DNA of a number of endogenous Vα segments (Figure 1). Thus, 1C6 T cells have a high degree of promiscuity in their TcRα repertoire.

**Figure 2:**
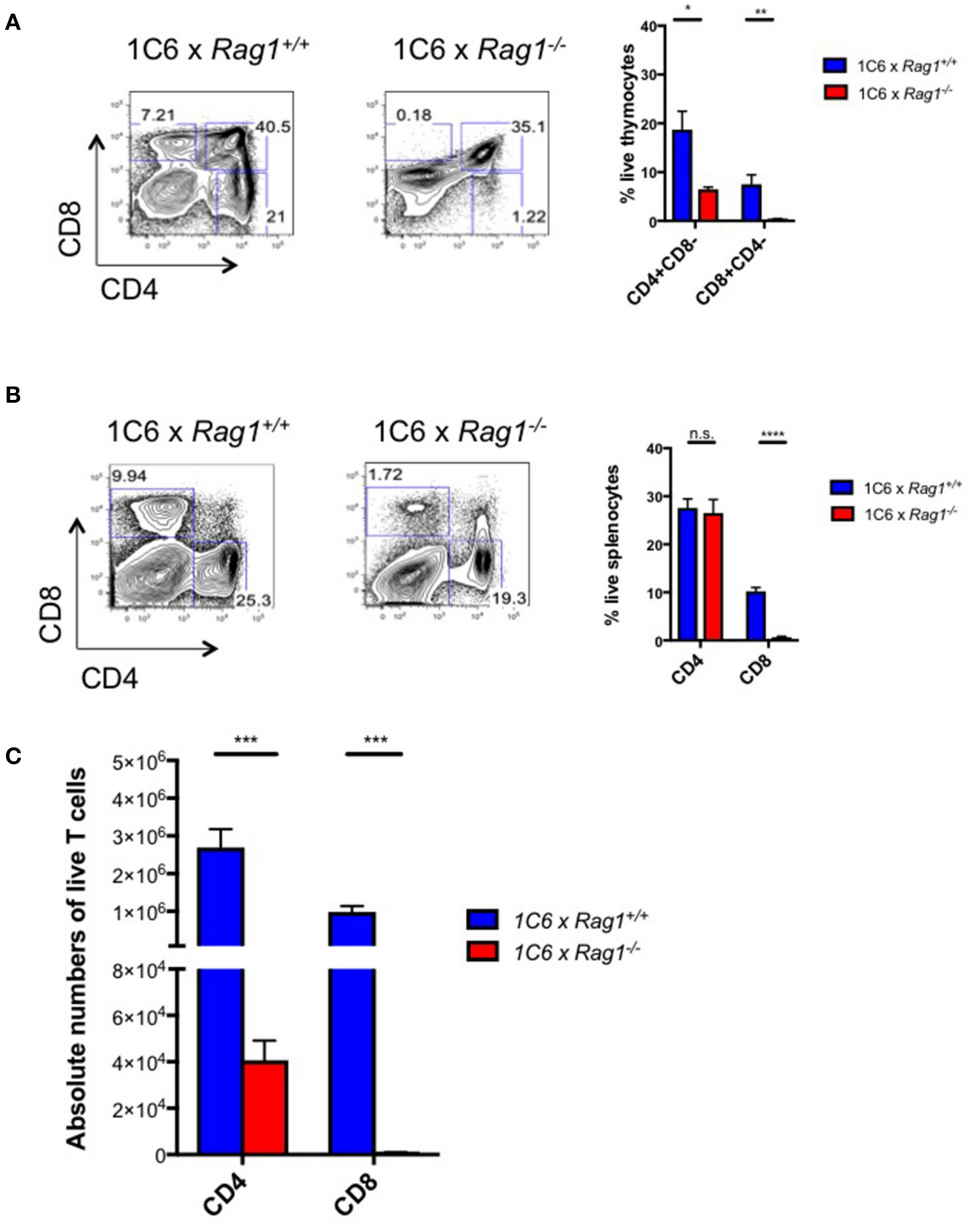
Peripheral T cell populations are dysregulated in 1C6 × *Rag1*^−/−^ mice due to defective negative selection. Thymic **(A)** and splenic **(B,C)** cell populations were enumerated from female 1C6 (*n* = 6) vs. 1C6 × *Rag1*^−/−^ (*n* = 6) mice. **(A)** The proportion of thymocytes expressing CD4 and/or CD8 was enumerated. The proportion **(B)** and absolute frequency **(C)** of splenic CD4^+^ and CD8^+^ T cells were assessed by flow cytometry. Graphs: *p < 0.05; **p < 0.01; ***p < 0.001; ****p < 0.0001, two-tailed *t*-test. n.s., not significant.

Next, we generated *1C6* × *Rag1*^−/−^ mice to test whether endogenously encoded TcR loci were functionally relevant to 1C6 CD8^+^ survival and function. These mice are unable to rearrange endogenous TcR loci but their T cells should bear transgenic 1C6 TcR. We found that in the absence of Rag1, there was a profound arrest at the double-positive stage of thymopoiesis (Figure 2A). This indicated that expression of endogenously derived TcR was essential for 1C6 thymocytes to escape negative selection. In the periphery, while the percentage of splenic CD4^+^ T cells was similar between 1C6 × *Rag1*^−/−^ and 1C6 mice, the frequency of CD8^+^ T cells was sharply reduced (Figure 2B). However, absolute numbers of both CD4^+^ and CD8^+^ T cells were dramatically diminished in 1C6 × *Rag1*^−/−^ mice (Figure 2C). Together, our data showed that allelic inclusion of endogenously rearranged TcR is both widespread and essential for proper development of the T cell repertoire in 1C6 mice.

### 1C6 × Rag1^−/−^ Mice Develop Severe EAE

**Figure 3:**
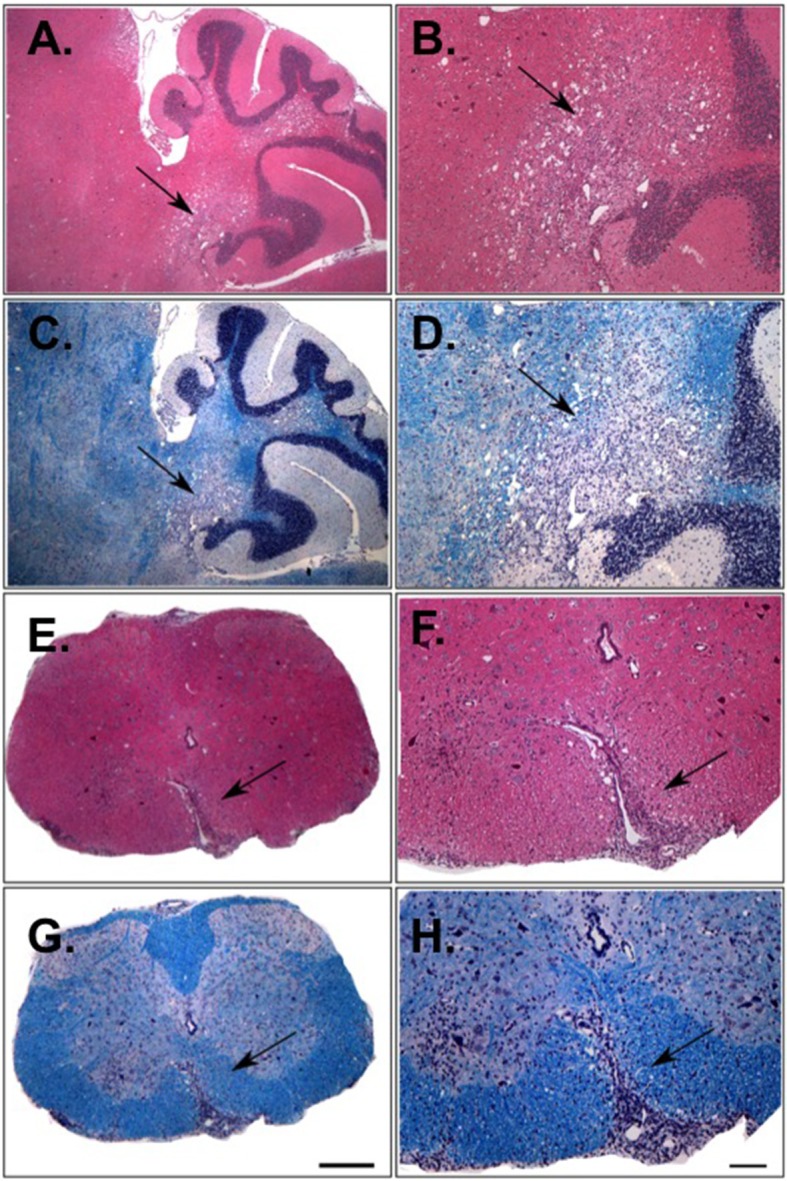
Histological analysis of the CNS of 1C6 × *Rag1*^−/−^ mice that develop spontaneous EAE. Cerebellar **(A–D)** and spinal cord **(E–H)** lesions from an 18-weeks old male 1C6 × *Rag1*^−/−^ mouse that spontaneously developed paralytic disease. H&E staining (**A,B,E,F**) was used to identify inflammatory foci, and Luxol fast blue was used to detect myelin **(C,D,G,H)**. Arrows indicate inflammatory damage. **(A,C,E,G)**, 10× magnification. **(B,D,F,H)**, 4× magnification. Scale bars, 100 μm. Representative of eight animals (6 female, 2 male).

Curiously, we also found that more than half (24/42) of 25-weeks old 1C6 × *Rag1*^−/−^ mice developed spontaneous paralytic disease (Table 1) characterized by immune cell infiltration and demyelination in the cerebellum and spinal cord (Figure 3). By contrast, no Rag1-sufficient 1C6 mice developed spontaneous disease in our colony (Table 1), in corroboration of previous findings that such symptoms occur only rarely in this strain (8).

**Table 1:** Incidence and severity of 1C6 × *Rag1*^−/−^ spontaneous EAE.

**Group #**	**Group ID**	**Sex**	**Incidence**	**Time of onset (weeks)**	**Mean max. score**
1	1C6	M	0/67^a^	n.a	n.a.
2	1C6	F	0/37^b^	n.a.	n.a.
3	1C6 × *Rag1^−/−^*	M	7/17^a^	17.3 ± 1.0	3.357 ± 0.5847
4	1C6 × *Rag1^−/−^*	F	17/25^b^	22.8 ± 5.5	3.647 ± 0.3707

*Unimmunized mice were followed until at least 25 weeks of age. Mean maximal score was calculated from mice that developed disease only. Time of onset and mean maximal score, mean ± s.e.m*.

a *p < 0.0001*;

b *p < 0.0001*.

*^a, b^ two-tailed Fisher's exact test with Bonferroni's correction. No significant differences in time of onset or mean maximal score were measured between male and female 1C6 × Rag1^−1^ mice*.

**Figure 4:**
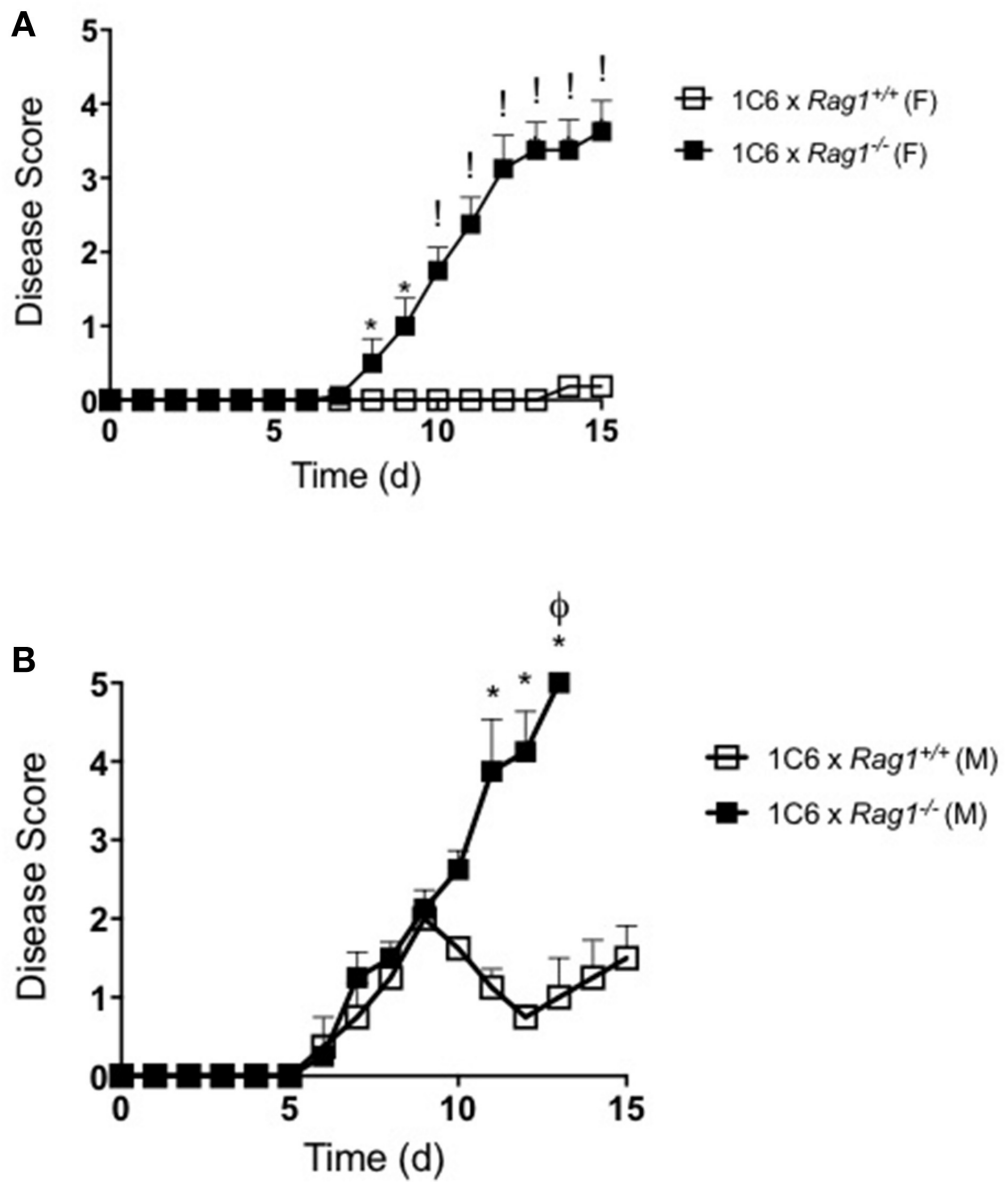
1C6 × *Rag1*^−/−^ mice develop severe MOG_[35−55]_-driven EAE. 1C6 (male, *n* = 4; female, *n* = 5) and 1C6 × *Rag1*^−/−^ (male, *n* = 4; female, *n* = 5) mice were actively immunized with MOG_[35−55]_ and were monitored for signs of EAE. **(A)** female, **(B)** male. *p < 0.05; !*p* < 0.001, two-tailed Mann-Whitney *U* test. ϕ indicates that all mice in the experimental group attained ethical endpoints. Representative of three immunizations.

To directly examine whether EAE in these animals was driven in an antigen-specific manner, we immunized 1C6 × *Rag1*^−/−^ and 1C6 × *Rag1*^+/+^ mice with MOG_[35−55]_. Both male and female 1C6 × *Rag1*^−/−^ mice developed severe, often fatal, EAE within 12–15 days. By contrast, immunized 1C6 × *Rag1*^+/+^ controls displayed mild disease, at best, during the same time frame (Figure 4). This is in line with previous findings that actively immunized NOD strain mice develop severe paralytic EAE (i.e., hind limb paralysis) only after 60 days (11, 12). Thus, despite having an extremely low frequency of peripheral T cells, 1C6 × *Rag1*^−/−^ mice were prone to develop CNS-targeted inflammation.

### 1C6 × Rag1^+/+^ CD4^+^ T Cells Ameliorate Disease in 1C6 × Rag1^−/−^ Mice

**Figure 5:**
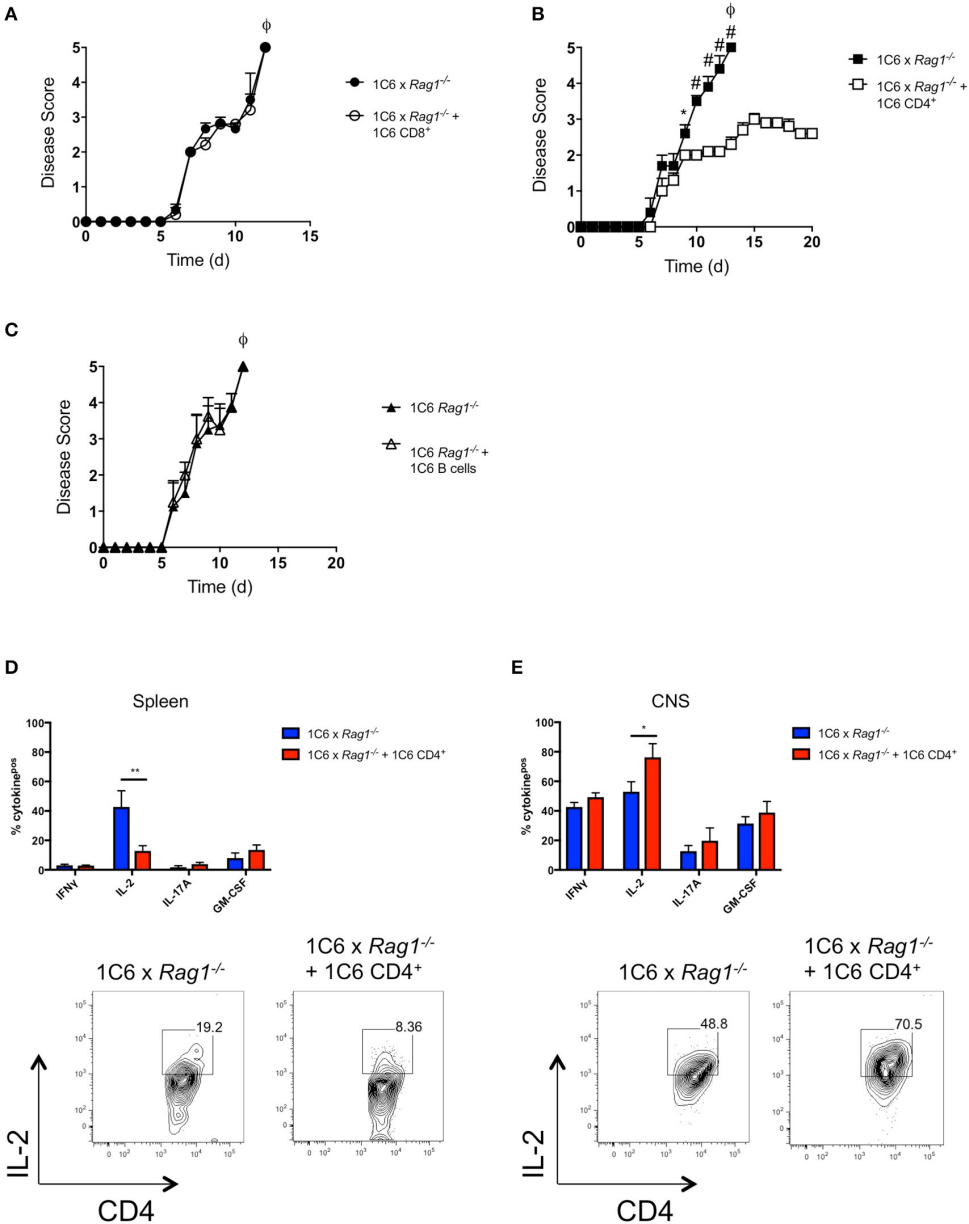
1C6 CD4^+^ T cells restrain CNS autoimmunity in 1C6 × *Rag1*^−/−^ mice. **(A)** Male 1C6 × *Rag1*^−/−^ mice were reconstituted (*n* = 5), or not (*n* = 5), with 2 × 10^6^ CD4^+^ T cells from unmanipulated male 1C6 mice. After 7 days, mice were actively immunized with MOG_[35−55]_ and were monitored for signs of EAE. Representative of 2 experiments. **(B)** Male 1C6 × *Rag1*^−/−^ mice were reconstituted (*n* = 5), or not (*n* = 5), with 2 × 10^6^ CD8^+^ T cells from unmanipulated male 1C6 mice. After 7 days, mice were actively immunized with MOG_[35−55]_ and were monitored daily for signs of EAE. *p < 0.05, two-tailed Mann-Whitney *U* test. ϕ indicates that all mice in the experimental group attained ethical endpoints. **(C)** Female 1C6 × *Rag1*^−/−^ mice were reconstituted (*n* = 4), or not (*n* = 4), with 2 × 10^6^ CD19^+^ B cells from unmanipulated 1C6 mice. After 7 days, mice were actively immunized with MOG_[35−55]_ and were monitored for signs of EAE. ϕ indicates that all mice in the experimental group attained ethical endpoints. *p < 0.05; #*p* < 0.01, two-tailed Mann-Whitney *U* test. ϕ indicates that all mice in the experimental group attained ethical endpoints. **(D,E)**. Spleen **(D)** or CNS-infiltrating **(E)** CD4^+^ T cells were isolated from immunized 1C6 × *Rag1*^−/−^ mice when they reached ethical endpoints (*n* = 9), or from 1C6 × *Rag1*^−/−^ (reconstituted with 1C6 CD4^+^) that were sacrificed in parallel (*n* = 4). T cells were assessed *ex vivo* for the indicated cytokines by flow cytometry. All data are gated on live CD4^+^ events. *p < 0.05; **p < 0.01, Sidak's multiple comparisons test after two-way ANOVA. Bottom plots, representative IL-2 expression from splenic **(D)** or CNS-infiltrating **(E)** CD4^+^ T cells.

We had observed that absolute numbers of CD4^+^ T cells were greatly reduced in 1C6 x *Rag1*^−/−^ mice (Figure 2C). This indicated that, despite the 1C6 TcR being class II-restricted, survival of 1C6 CD4^+^ T cells was partially dependent on endogenous TcR rearrangement. Further, in light of the fact that 1C6 CD8^+^ T cells could not ameliorate the severe disease observed in 1C6 × *Rag1*^−/−^ mice, it raised the possibility that 1C6 CD4^+^ T cells may themselves contain a regulatory population. To address this, we asked whether passive transfer of 1C6 CD4^+^ T cells could rescue 1C6 × *Rag1*^−/−^ mice from severe MOG_[35−55]_-driven EAE. We therefore passively transferred Rag-sufficient 1C6 T cells to 1C6 × *Rag1*^−/−^ mice, and 7 days later, immunized them in parallel with 1C6 × *Rag1*^−/−^ mice that did not receive 1C6 CD4^+^ T cells. As expected, 1C6 × *Rag1*^−/−^ rapidly developed fatal EAE. By contrast, 1C6 × *Rag1*^−/−^ mice receiving Rag1-sufficient 1C6 CD4^+^ T cells did not develop fatal disease when monitored for 20 days (Figure 5A). As we had also found that CD8^+^ T cells were profoundly diminished in 1C6 × *Rag1*^−/−^ mice, and as substantial evidence exists that in certain contexts, CD8^+^ regulatory T cells (T_reg_) can suppress inflammation in MS and EAE (13), we wanted to rule out the possibility that exacerbated disease in 1C6 × *Rag1*^−/−^ mice was due to a lack of a CD8^+^ T_reg_. To address this, we infused 1C6 × *Rag1*^−/−^ mice with 1C6 × *Rag1*^+/+^ CD8^+^ T cells and immunized them with MOG_[35−55]_ after 7 days. We observed no differences in disease severity between these animals and unmanipulated 1C6 × *Rag1*^−/−^ mice (Figure 5B). Further, we were able to rule out a potential role for regulatory B cells (14) in controlling disease severity, as prior transfer of 1C6 strain B cells to 1C6 × *Rag1*^−/−^ mice did not protect them from severe EAE (Figure 5C). Taken altogether, our data showed that the exacerbated disease seen in 1C6 × *Rag1*^−/−^ mice resulted from the absence of a CD4^+^ T cell subpopulation normally resident in 1C6 mice.

We next examined *ex vivo* cytokine production from the CD4^+^ T cell compartment of 1C6 × *Rag1*^−/−^ mice that had been reconstituted with 1C6 × *Rag1*^+/+^ CD4^+^ T cells prior to immunization. We therefore repeated our 1C6 CD4^+^ passive transfer protocol in 1C6 × *Rag1*^−/−^ mice, followed by MOG_[35−55]_ immunization. Spleen and CNS-infiltrating T cells were isolated from unreconstituted, immunized, 1C6 × *Rag1*^−/−^ mice when they reached ethical endpoints and from 1C6 CD4^+^ T cell-reconstituted counterparts that we sacrificed in parallel. In the spleen, IL-2 was selectively decreased in 1C6 × *Rag1*^−/−^ mice that received 1C6 CD4^+^ T cells; no differences were observed in the production of IFNγ, IL-17, GM-CSF, or TNFα (Figure 5D). Curiously and in contrast, among CNS-infiltrating CD4^+^ T cells, IL-2 production was selectively increased in mice that had been infused with 1C6 × *Rag1*^+/+^ CD4^+^ T cells (Figure 5E).

### CNS-Infiltrating FoxP3^+^CD4^+^ Are Absent in the CNS of 1C6 × Rag1^−/−^ EAE Mice

**Figure 6:**
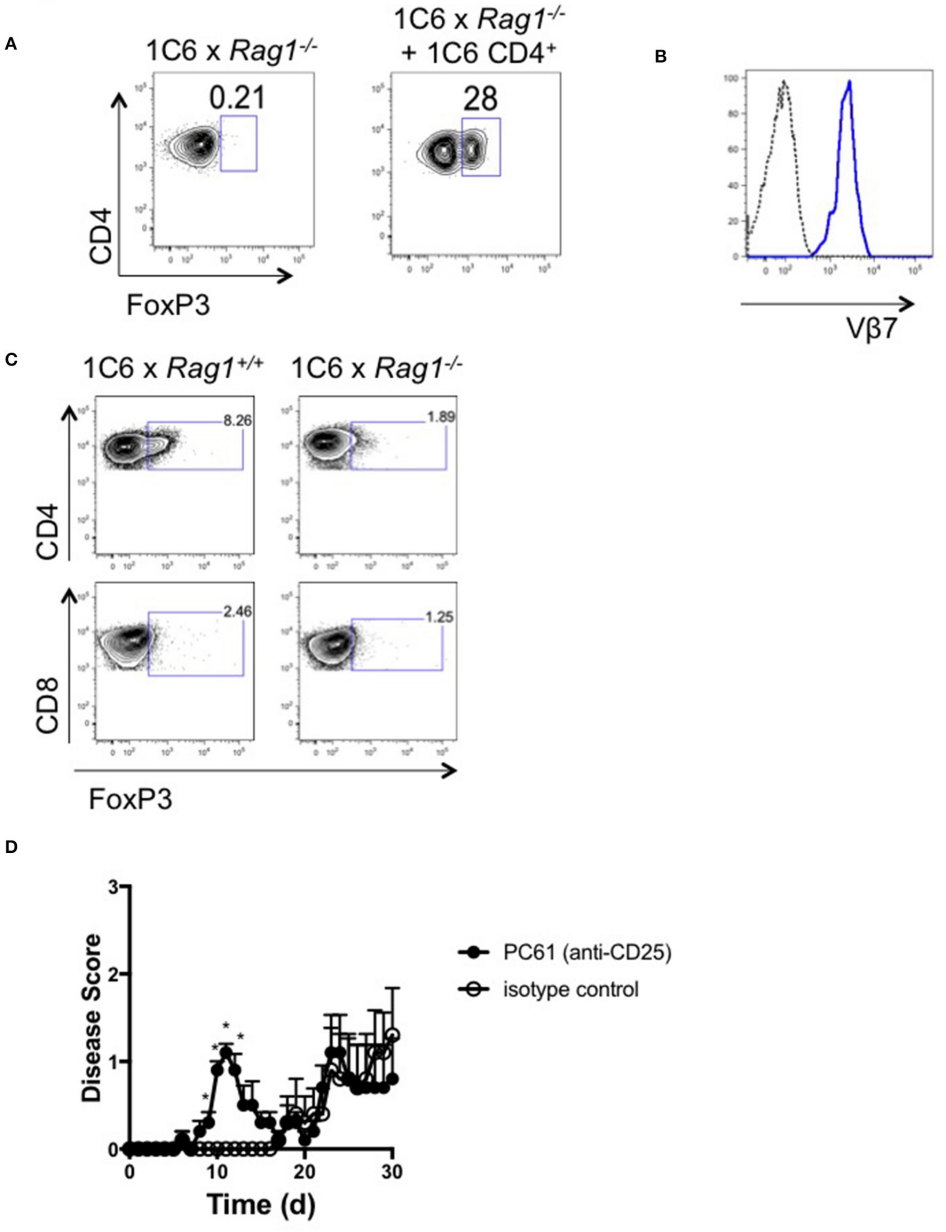
FoxP3^+^ CD4^+^ T cells are absent from the CNS of 1C6 × *Rag1*^−/−^ mice with EAE. **(A,B)**. Female 1C6 × *Rag1*^−/−^ mice were reconstituted, or not, with 2 × 10^6^ CD4^+^ T cells from unmanipulated 1C6 mice. After 14 days, mice were actively immunized with MOG_[35−55]_. At experimental endpoints, CNS-infiltrating CD4^+^ T cells were assessed by flow cytometry for expression of FoxP3 **(A)**. Representative data, *n* = 4 each group. **(B)** Vβ7 expression (blue line) on CNS-infiltrating CD4^+^FoxP3^+^ T cells from a 1C6 × *Rag1*^−/−^ mouse reconstituted with 1C6 CD4^+^ T cells. Dotted line, FMO control. **(C)** Female 1C6 and 1C6 × *Rag1*^−/−^ mice were actively immunized with MOG_[35−55]_. Lymph node T cells were isolated at the peak of disease and assessed by flow cytometry for FoxP3 expression. Gated on CD4^+^ (top) or CD8^+^ (bottom) events. Representative of 5 mice each. **(D)** Male 1C6 mice were treated with Treg-depleting anti-CD25 antibody (PC61) or with isotype control on days−11,−9, and−7, and were immunized with MOG_[35−55]_ on d0. *n* = 5, both groups. *p < 0.05 on individual days, Mann-Whitney *U* test.

FoxP3^+^CD4^+^ T_reg_ (15) control both onset and severity of EAE (16) and their survival is dependent on IL-2 (17). Given the fact that 1C6 × *Rag1*^+/+^ CD4^+^ T cells rescued 1C6 × *Rag1*^−/−^ mice from severe EAE, as well as the observed anomalies in IL-2 production in the presence or absence of Rag-sufficient 1C6 T cells, we next wanted to examine whether 1C6 CD4^+^ T cell infusion could drive T_reg_ expansion. While absent from immunized 1C6 × *Rag1*^−/−^ mice at experimental endpoints, 1C6 CD4^+^ T cell reconstitution increased the frequency of FoxP3^+^CD4^+^ T cells in the CNS (Figure 6A). CNS-infiltrating T_reg_ were largely Vβ7^+^ (Figure 6B), indicating that endogenous TcR rearrangement was restricted to the α-chain in these cells.

It was possible that the expansion of 1C6-origin CD4^+^ T_reg_ in 1C6 × *Rag1*^−/−^ mice was a homeostatic response to a lack of this population in 1C6 × *Rag1*^−/−^ mice. To rule out this possibility, we assessed the presence of CD4^+^ T_reg_ in actively immunized 1C6 animals. FoxP3^+^CD4^+^ T cells were identified in the CNS of 1C6 mice and were, as expected, absent in 1C6 × *Rag1*^−/−^ mice (Figure 6C). CD8^+^FoxP3^+^ cells were not seen in either genotype at an appreciable frequency. Thus, our data show that CD4^+^FoxP3^+^ T_reg_ are critical to controlling EAE severity in the 1C6 strain, and that their development is crucially dependent on the arrangement of endogenous TcRα.

Given our data pointed toward an important role for T_reg_ in suppressing immune control of CNS autoimmunity in NOD-EAE, we next wanted to assess the effects of T_reg_ depletion in immunized 1C6 mice. We treated mice with 3 successive doses of anti-CD25 monoclonal antibody (clone PC61), which depletes T_reg_, or with isotype control, and subsequently immunized them with MOG_[35−55]_ (18). PC61-treated mice developed disease of earlier onset than that observed in control-treated animals (8.4 days ± 0.7 vs. 18.6 ± 3.5, *p* < 0.021; Figure 6D), indicating an important role for T_reg_ in regulating the initiation of autoimmune symptoms in NOD EAE.

## Discussion

Our data indicate that 1C6 T cells can express multiple endogenous Vα chains, and that endogenous TcR rearrangement is critical for the selection of CD4^+^ FoxP3^+^ T_reg_ that mitigate disease in Rag1-sufficient 1C6 mice. Few CD4^+^ or CD8^+^ single-positive thymocytes were detected in 1C6 × *Rag1*^−/−^ thymi, indicating that the 1C6 TcR is highly prone to negative selection. These observations are in contrast to what is known about other self-reactive TcR-Tg strains. T cells with a myelin basic protein (MBP)_[1−9]_-specific TcR were thymically selected even when crossed to a *Rag1*^−/−^ background; further, numbers of splenic CD4^+^ T cells were similar between Rag1-sufficient (“T/R^+^”) and Rag1-deficient (“T/R-”) mice (9). Similarly, the proteolipid protein (PLP)_139−151_-specific “5B6” TcR successfully escapes negative selection on a Rag-deficient background (10). Our data are striking when one considers that the parental 1C6 T cell clone was isolated from the peripheral lymphoid tissue of an immunized WT NOD mice, and thus by definition escaped thymic negative selection in the thymus. TcR signal strength to self is thought to dictate whether a thymocyte passes the single-positive phase, with strongly self-reactive clones being negatively selected. The 1C6 TcR is thus one that is likely to be highly self-reactive and rarely present in the periphery of WT mice. In the future, it will be interesting to compare the affinity of 1C6 to MOG_[35−55]_/class II to that of other MOG_[35−55]_-specific TcR such as 2D2. Further, while our data indicate that 1C6 T cells are highly promiscuous in their expression of Vα at a population level, we do not know whether individual T cells in these mice escape negative selection by expressing both the 1C6 TcR as well as a second TcR consisting of Vβ7 paired to an endogenous Vα, or whether they lost 1C6 TcR completely and express only endogenous TcR. Unfortunate, the current lack of antibodies against the full spectrum of Vα makes it challenging to distinguish between these possibilities using flow cytometry. In the future, however, it may be possible to use next-generation sequencing techniques to characterize TcRα/β pairing at the single cell level.

Despite having few circulating T cells, 1C6 × *Rag1*^−/−^ mice developed extremely severe EAE. By contrast, EAE in NOD strain mice develops relatively slowly, with advanced paralytic disease observed only after 60 days. Disease in actively immunized 1C6 × *Rag1*^−/−^ was characterized by augmented expression of the T cell growth factor IL-2 from peripheral CD4^+^ T cells. Thus, 1C6 × *Rag1*^−/−^ T cells are highly autoreactive, in line with their propensity to be negatively selected. Indeed, while transfer of Rag-sufficient 1C6 CD4^+^ T cells to 1C6 × *Rag1*^−/−^ saved these mice from fulminant CNS autoimmunity, they retained a significant amount of disability despite the fact that the FoxP3^+^ T_reg_ niche was reconstituted, suggesting again that the 1C6 TcR is itself highly pathogenic.

Interestingly, there were no differences in production of IFNγ or IL-17 between CNS infiltrating CD4^+^ T cells from immunized 1C6 × *Rag1*^−/−^, vs. 1C6 × *Rag1*^−/−^ reconstituted with 1C6 CD4^+^ T cells, suggesting that loss of endogenous TcR rearrangement did not skew the immune response toward either Th1 or Th17. This, together with the fact that the severe disease phenotype of 1C6 × *Rag1*^−/−^ mice could be partially rescued by the presence of Rag-sufficient 1C6 CD4^+^, but not CD8^+^, T cells, led us to ask whether an absence of T_reg_ may explain the phenotype. Indeed, FoxP3^+^ CD4^+^ T_reg_ were absent from immunized 1C6 × *Rag1*^−/−^ mice, indicating that their severe disease was at least in part due to a loss of this subpopulation. 1C6 T_reg_ were Vβ7^+^, indicating that TcR rearrangement in these cells occurred chiefly for the α-chain. Strikingly, *in vivo* depletion of CD25^+^ T_reg_ accelerated the onset of symptoms in 1C6 mice, indicating that T_reg_ control the initiation of autoimmune reactivity in this model. Depleted mice did not progress to fulminant EAE; it is possible that CD25^−^ T_reg_, which play important roles in immunoregulation (19) and which would be preserved in our protocol, might also contribute to the control of CNS autoimmunity in Rag1-sufficient animals.

Tonegawa et al. previously reported that Rag-deficient, MBP_[1−9]_-specific, “T/R^−^” mice develop spontaneous EAE with 100% frequency (9). Disease in “T/R^−^” mice was rescued by the transfer of Rag-sufficient Tg “T/R^+^” (20) or wildtype (21) T cells. These cells were later shown to be CD4^+^CD25^+^ (22) and presumably FoxP3^+^ T_reg_. Rag deficiency has additionally been shown to exacerbate EAE in two Tg strains featuring a humanized TcR specific for myelin antigen (23, 24). Thus, our data, and that of others, indicate that functionally important T_reg_ can be present in the periphery, even when one skews the repertoire toward an autoreactive TcR specificity derived from an inflammatory effector T cell clone. This raises the question of whether allelic inclusion generates *de novo* TcR that drive T_reg_ generation, possibly due to the strength of their interactions with self-Ag in the thymus. This would be exciting, as it would indicate that there are specific TcR rearrangements that we could identify that promote peripheral tolerance to CNS antigen via the development of T_reg_. Evidence supporting this model comes from Bautista and colleagues, who found that when introduced into wildtype thymi, donor thymocytes expressing a T_reg_-specific TcR could give rise to FoxP3^+^ cells. By contrast, thymocytes expressing TcR to non-self Ag did not have this effect (25). Leung and colleagues additionally showed a higher frequency of donor-derived FoxP3^+^ cells when T_reg_-specific TcR-expressing thymi were introduced to recipient mice. However, this latter study also found that the percentage of donor-derived FoxP3^+^ T cells in these animals did not exceed the frequency of T_reg_ seen in a normal mouse. Thus, while specific TcR may indeed preferentially give rise to FoxP3^+^ T_reg_, it is also likely that the development of these cells is regulated homeostatically. It remains to be seen whether 1C6-origin T_reg_ recognize MOG_[35−55]_ or other Ag via their endogenous TcR. It must also be noted that endogenous TcR rearrangement is not a universally critical mechanism of peripheral tolerance in TcR-Tg strains, as 2D2 mice do not appear to develop worsened disease when crossed to a *Rag2*^−/−^ background (26). The capacity of background-specific MHC to present antigen is also likely to play a role in the development of T_reg_ in these strains.

This work describes the requirement for endogenous TcR rearrangement for appropriate T cell development in the 1C6 transgenic strain. Peripheral T cells are profoundly reduced when expression of the transgenic TcR is enforced on a *Rag1*^−/−^ background. Despite the reduction in T cells, 1C6 × *Rag1*^−/−^ mice are susceptible to severe EAE that is at least partially due to an absence of CD4^+^, but not CD8^+^, T_reg_. These findings underscore the clonal complexity of T cell-driven responses in the context of autoimmunity.

## Materials and Methods

### Animals and EAE Induction

1C6 mice were a kind gift of Dr. Vijay Kuchroo (Boston, MA) and were maintained in our facility. *Rag1*^−/−^ NOD strain mice were obtained from JAX and were crossed to the 1C6 strain in our facility. The sex of the mice used in each experiment is indicated in the Figure legends and in Table 1. Mice were monitored bi-weekly for initial signs of spontaneous paralytic disease. Mice that developed spontaneous disease were monitored daily. Active immunization was conducted at 10–12 weeks of age similar to as described previously (27). Briefly, they were immunized with 200 μg MOG_[35−55]_ in 100 μL of complete Freund's adjuvant (Difco) supplemented with 500 μg *M. tuberculosis* extract (Difco). Mice received 200 ng pertussis toxin (List Biological Labs) on d0 and d2. For lymphocyte rescue experiments, 1C6 × *Rag1*^−/−^ mice were passively transferred with 2 × 10^6^ purified splenic 1C6 CD4^+^ T, CD8^+^ T, or CD19^+^ B cells. Rescue mice were then rested for 7 days prior to immunization in parallel with controls. For T_reg_ depletion protocol, mice received 3 injections 100 μg of PC61 or isotype control antibody, each separated by 48 h (18). Seven days after last injection, mice were immunized as described above. All EAE mice were weighed and assessed for disease symptoms daily for up to 30 days, using an established semi-quantitative scale that we have used previously (28): 0, no disease; 1, decreased tail tone; 2, hind limb weakness or partial paralysis; 3, complete hindlimb paralysis; 4, front and hind limb paralysis; 5, dead or having attained ethical endpoints. Moribund mice were euthanized with 24 h if they did not show improvement. “Disease onset” is defined as being within 24 h of the first signs of clinical symptoms.

### Antibodies

Flow cytometry monoclonal Abs (mAbs) against mouse antigens were obtained from eBioscience (CD4, clone RM4-5; CD8α, clone 53–6.7; CD19, clone eBio1D3; IFNγ, clone XMG1.2; GM-CSF, clone MP1-22E9), BioLegend (Vβ7, clone TR3-10; IL-17A, clone TC11-18H10.1; FoxP3, clone FJK-16S) or BD (IL-2, clone JES6-5H4); anti-CD25 for *in vivo* depletion, as well as rIgG1 isotype control, were obtained from BioXcell.

### T Cell Purification

Mononuclear cell preparations were obtained from mouse spleens of donor mice. CD4^+^ T cells, CD8^+^ T cells and CD19^+^ B cells were enriched using the appropriate MACS beads (Miltenyi). They were then incubated with the appropriate (CD4, CD8, CD19) fluorochrome-labeled antibodies and purified using high-speed cell sorting (FACSAria, BD).

### TcR Vα PCR

CD8^+^ T cells were purified from 1C6 mouse spleen using high-speed cell sorting, and genomic DNA was isolated using DNA Miniprep kit (Bio Basic Canada). PCR amplification of different regions of TCRAV was carried out using a Bio-Rad C1000 Touch thermocycler. In each PCR reaction, 20 ng of genomic DNA was added to 20 μL of reaction mixture consisting of 1X Accustart II PCR super mix (Quanta Bio). Allele-specific primers were added at 0.2 μmol/ reaction. Each PCR reaction consisted 35 cycles of 2 min of denaturation at 95°C, 30 s 95°C, annealing at 58°C, and 1 min of extension at 72°C, and a final extension step of 5 min at 72°C. At the end of the reaction, the PCR product was mixed with 2 μL of loading dye (200 g/L sucrose, 0.05 g/L bromphenol blue) and was then separated on 1% agarose gel. Primer sequences were designed using PrimerQuest software (IDT) and were as follows: (1) *TRAV1.2*, 5′-CAGCCTGCCAAATTGATGTC-3′, 5′-ACAGAGGTATGAGGCAGAGT-3′, 260 bp amplicon; (2) *TRAV2*, 5′-CTTGCCAAGACCACCCA-3′, 5′-GTCAGTCACAATGCAGTAATACAC-3′, 272 bp; (3) *TRAV3*, 5′-CCCTCCTCACCTGAGTGT-3′, 5′-GATGGGCAGCTGTGAGG-3′, 224 bp; (4) *TRAV3.2*, 5′-GAGAGCAGGTGGAGCATTG-3′, 5′-ACTTGCTGCACAGAAGTACA-3′, 272 bp; (5) *TRAV4.3*, 5′-GTGCAGATTTGCTGTGAGTTG-3′, 5′-CCTCAGCAGCACAGAAGTAA-3′, 464 bp; (6) *TRAV5.1*, 5′-TGGAACAGCTCCCTTCCT-3′, 5′-ACTTGCTGAGCAGAAGTAGATG-3′, 269 bp; (7) *TRAV6.1*, 5′-GGAGACTCAGTGACCCAGAT-3′, 5′-CACCCAGAACACAGTAGTACA-3′, 280 bp; (8) *TRAV7.2*, 5′-AACAGAAGGTACAGCAGAGC-3′, 5′-TGCTCACTGCACAGAAGTAG-3′, 276 bp; (9) *TRAV8.1*, 5′-AGTCAACTAGCAGAAGAGAATCC-3′, 5′CATCAGTAGCACAGAAGTACACA-3′, 277 bp; (10) *TRAV9*, 5′-ATCTCGTTCCTCGGGATACA-3′, 5′-GCTCACAGCACAGAAGTACA-3′, 446 bp; (11) *TRAV10*, 5′-TCCCTTCACACTGTATTCCTATTC-3′, 5′-GGAGAATCGTTTGGCTTTCTTATC-3′, 430 bp; (12) *TRAV11*, 5′-TGGTCTTGTGGCTGCATTAT GTGCTGTGCTTAGCATCTTTATC-3′, 466 bp; (13) *TRAV13*, 5′-GTTTGCTGTGAGTCTGGGT CACAGAGATAAGTGCCTGAGTC-3′, 458 bp.

### Histopathology

Mice were euthanized and perfused first with cold PBS and then with 4% paraformaldehyde (PFA) through the left cardiac ventricle. Brains and spinal cords were dissected from the skull and spinal column, respectively. Tissues were incubated for 24 h in 4% PFA at 4°C and then for a minimum of 48 h in PBS before being embedded in paraffin. Five-micrometer thick sections of the brain and spinal cord were stained with hematoxylin & eosin (H&E) or Luxol fast blue for myelin (29). Images were taken using Nova Prime software (Bioquant Image Analysis Corporation) with a high-resolution QICAM fast color 1,394 camera (1,392 × 1,040 pixels; OImaging) installed on a Nikon Eclipse 80i microscope. Images were taken at 4× and 10× magnification and were calibrated with a microscope micrometer calibration ruler using FiJi software (NIH).

### CNS Mononuclear Cell Isolation

Mice were euthanized and perfused with cold PBS administered through the left cardiac ventricle. Brains and spinal cords were dissected from the skull and spinal column, respectively. CNS tissue were homogenized using a PTFE Tissue Grinder (VWR) and were incubated for 30 min at 37°C in homogenization solution (HBSS containing 4 ng mL-1 liberase and 25 ng mL-1 DNase). Homogenate was filtered through a 70-μm cell strainer, resuspended in 35% Percoll (GE Healthcare) and centrifuged. Mononuclear cells were collected, washed and prepared for flow cytometric analysis.

### Flow Cytometry

Staining for cell surface markers was carried out for 30 min at 4°C. Prior to incubation with mAbs against cell surface markers, cells were incubated for 10 min in the presence of Fc Block (BD Biosciences) to prevent non-specific Ab binding to cells. For analysis of intracellular cytokine expression, cells were first cultured for 4 h in the presence of 50 ng mL^−1^ phorbol 12-myristate 13-acetate (Sigma-Aldrich), 1 μM ionomycin (Sigma-Aldrich) and GolgiStop (1 μL per mL culture; BD Biosciences). Cells were subsequently incubated with Fc Block (BioLegend) and fluorescent cell surface marker Abs and were then fixed and permeabilized using Fixation and Perm/Wash buffers (eBioscience). They were then stained with fluorescent Abs against intracellular markers. FoxP3 Fix/Perm and Perm buffers (BioLegend) were used for FoxP3 staining. Flow cytometry data were collected using a LSRII flow cytometer (BD Biosciences) and were analyzed using FlowJo software (Treestar). Dead cells were excluded from analysis on the basis of positivity for Fixable Viability Dye (eBioscience). Gates were set on the basis of fluorescence minus one controls.

### Statistical Analysis

Two-tailed comparisons were made in all cases. For EAE data, *t*-test was used to analyze mean week of onset. Mann-Whitney *U* test was used to analyze mean maximal severity and severity on individual days. Fisher's exact test was used to analyze disease incidence and mortality incidence with Bonferroni's correction being applied. *Ex vivo* production of cytokines was assessed by two-way ANOVA followed by Sidak's multiple comparisons test. All statistical analyses were performed using Prism (GraphPad), with the exception of Fisher's exact test (QuickCalc online tool, https://www.graphpad.com/quickcalcs/contingency1.cfm; GraphPad). Error bars represent standard error (s.e.m.).

## Data Availability Statement

The datasets generated for this study are available on request to the corresponding author.

## Ethics Statement

All experimental protocols and breedings were approved by the Animal Protection Committee of Laval University (2017-037-2 and 2017-090-2).

## Author Contributions

AY directed the project and conducted experiments. PI, JB, IA, and MB conducted experiments. BM and SL conducted histopathological analyses. AY, PI, ST, and AA assisted with writing the manuscript. MR supervised the project and wrote the manuscript.

### Conflict of Interest

MR has performed educational activities for Biogen Canada and is the lead investigator on a research contract with Remedy Pharmaceuticals. These activities are unrelated to the work presented in this manuscript. The remaining authors declare that the research was conducted in the absence of any commercial or financial relationships that could be construed as a potential conflict of interest.

## References

[1] Haghikia A Hohlfeld R Gold R Fugger L. Therapies for multiple sclerosis: translational achievements and outstanding needs. Trends Mol Med. (2013) 19:309–19. 10.1016/j.molmed.2013.03.004 23582699

[2] Vargas DL Tyor WR. Update on disease-modifying therapies for multiple sclerosis. J Investig Med. (2017) 65:883–91. 10.1136/jim-2016-000339 28130412

[3] Rangachari M Kuchroo VK. Using EAE to better understand principles of immune function and autoimmune pathology. J Autoimmun. (2013) 45:31–9. 10.1016/j.jaut.2013.06.008 23849779 PMC3963137

[4] Bettelli E. Building different mouse models for human MS. Ann N Y Acad Sci. (2007) 1103:11–18. 10.1196/annals.1394.021 17376825

[5] Basso AS Frenkel D Quintana FJ Costa-Pinto FA Petrovic-Stojkovic S Puckett L. Reversal of axonal loss and disability in a mouse model of progressive multiple sclerosis. J Clin Invest. (2008) 118:1532–43. 10.1172/JCI33464 18340379 PMC2267014

[6] Farez MF Quintana FJ Gandhi R Izquierdo G Lucas M Weiner HL. Toll-like receptor 2 and poly(ADP-ribose) polymerase 1 promote central nervous system neuroinflammation in progressive EAE. Nature Immunol. (2009) 10:958–64. 10.1038/ni.1775 19684606 PMC2746562

[7] Mayo L Trauger SA Blain M Nadeau M Patel B Alvarez JI. Regulation of astrocyte activation by glycolipids drives chronic CNS inflammation. Nat Med. (2014) 20:1147–56. 10.1038/nm.3681 25216636 PMC4255949

[8] Anderson AC Chandwaskar R Lee DH Sullivan JM Solomon A Rodriguez-Manzanet R. A transgenic model of central nervous system autoimmunity mediated by CD4^+^ and CD8^+^ T and B cells. J Immunol. (2012) 188:2084–92. 10.4049/jimmunol.1102186 22279107 PMC3288950

[9] Lafaille JJ Nagashima K Katsuki M Tonegawa S. High incidence of spontaneous autoimmune encephalomyelitis in immunodeficient anti-myelin basic protein T cell receptor transgenic mice. Cell. (1994) 78:399–408. 10.1016/0092-8674(94)90419-7 7520367

[10] Waldner H Whitters MJ Sobel RA Collins M Kuchroo VK. Fulminant spontaneous autoimmunity of the central nervous system in mice transgenic for the myelin proteolipid protein-specific T cell receptor. Proc Natl Acad Sci USA. (2000) 97:3412–7. 10.1073/pnas.97.7.3412 10737797 PMC16253

[11] Encinas JA Wicker LS Peterson LB Mukasa A Teuscher C Sobel R. QTL influencing autoimmune diabetes and encephalomyelitis map to a 0.15-cM region containing Il2. Nat Genet. (1999) 21:158–60. 10.1038/5941 9988264

[12] Quintana FJ Jin H Burns EJ Nadeau M Yeste A Kumar D. Aiolos promotes TH17 differentiation by directly silencing Il2 expression. Nat Immunol. (2012) 13:770–7. 10.1038/ni.2363 22751139 PMC3541018

[13] Sinha S Boyden AW Itani FR Crawford MP Karandikar NJ. CD8^+^ T-cells as immune regulators of multiple sclerosis. Front Immunol. (2015) 6:619. 10.3389/fimmu.2015.00619 26697014 PMC4674574

[14] Pennati A Ng S Wu Y Murphy JR Deng J Rangaraju S. Regulatory B cells induce formation of IL-10-expressing T cells in mice with autoimmune neuroinflammation. J Neurosci. (2016) 36:12598–610. 10.1523/JNEUROSCI.1994-16.2016 27821578 PMC5157105

[15] Fontenot JD Gavin MA Rudensky AY. Foxp3 programs the development and function of CD4^+^CD25^+^ regulatory T cells. Nat Immunol. (2003) 4:330–6. 10.1038/ni904 12612578

[16] O'Connor RA Anderton SM. Foxp3+ regulatory T cells in the control of experimental CNS autoimmune disease. J Neuroimmunol. (2008) 193:1–11. 10.1016/j.jneuroim.2007.11.016 18077005

[17] D'Cruz LM Klein L. Development and function of agonist-induced CD25^+^Foxp3^+^ regulatory T cells in the absence of interleukin 2 signaling. Nat Immunol. (2005) 6:1152–9. 10.1038/ni1264 16227983

[18] Akirav EM Bergman CM Hill M Ruddle NH Depletion of CD4^+^CD25^+^ T cells exacerbates experimental autoimmune encephalomyelitis induced by mouse, but not rat, antigens. J Neurosci Res. (2009) 87:3511–9. 10.1002/jnr.21981 19125411 PMC4429897

[19] Coleman MM Finlay CM Moran B Keane J Dunne PJ Mills KHG. The immunoregulatory role of CD4^+^ FoxP3^+^ CD25^−^ regulatory T cells in lungs of mice infected with Bordetella pertussis. FEMS Immunol Med Microbiol. (2012) 64:413–24. 10.1111/j.1574-695X.2011.00927.x 22211712

[20] Olivares-Villagómez D Wang Y Lafaille JJ. Regulatory CD4^+^ T cells expressing endogenous T cell receptor chains protect myelin basic protein-specific transgenic mice from spontaneous autoimmune encephalomyelitis. J Exp Med. (1998) 188:1883–94. 10.1084/jem.188.10.1883 9815266 PMC2212402

[21] Van de Keere F Tonegawa S. CD4^+^ T cells prevent spontaneous experimental autoimmune encephalomyelitis in anti-myelin basic protein T cell receptor transgenic mice. J Exp Med. (1998) 188:1875–82. 10.1084/jem.188.10.1875 9815265 PMC2212404

[22] Matejuk A Buenafe AC Dwyer J Ito A Silverman M Zamora A. Endogenous CD4^+^BV8S2^−^ T cells from TG BV8S2+ donors confer complete protection against spontaneous experimental encephalomyelitis. (Sp-EAE) in TCR transgenic, RAG-/- mice. J Neurosci Res. (2003) 71:89–103. 10.1002/jnr.10450 12478617

[23] Ellmerich S Takacs K Mycko M Waldner H Wahid F Boyton RJ. Disease-related epitope spread in a humanized T cell receptor transgenic model of multiple sclerosis. Eur J Immunol. (2004) 34:1839–48. 10.1002/eji.200324044 15214032

[24] Madsen LS Andersson EC Jansson L krogsgaard M Andersen CB Engberg J. A humanized model for multiple sclerosis using HLA-DR2 and a human T-cell receptor. Nat Genet. (1999) 23:343–7. 10.1038/15525 10610182

[25] Bautista JL Lio C-WJ Lathrop SK Forbush K Liang Y Luo J. Intraclonal competition limits the fate determination of regulatory T cells in the thymus. Nat Immunol. (2009) 10:610–7. 10.1038/ni.1739 19430476 PMC2756247

[26] Krishnamoorthy G Saxena A Mars LT Domingues HS Mentele R Ben-Nun A. Myelin-specific T cells also recognize neuronal autoantigen in a transgenic mouse model of multiple sclerosis. Nat Med. (2009) 15:626–32. 10.1038/nm.1975 19483694

[27] Rangachari M Zhu C Sakuishi K Xiao S Karman J Chen A. Bat3 promotes T cell responses and autoimmunity by repressing Tim-3–mediated cell death and exhaustion. Nat Med. (2012) 18:1394–400. 10.1038/nm.2871 22863785 PMC3491118

[28] Boivin N Baillargeon J Doss PMIA Roy A-P Rangachari M. Interferon-β suppresses murine Th1 cell function in the absence of antigen-presenting cells. PLoS ONE. (2015) 10:e0124802. 10.1371/journal.pone.0124802 25885435 PMC4401451

[29] Bauer J Lassmann H. Neuropathological techniques to investigate central nervous system sections in multiple sclerosis. Methods Mol Biol. (2016) 1304:211–29. 10.1007/7651_2014_151 25520281

